# PREDICTORS OF LONGITUDINAL COGNITIVE DECLINE AMONG COMMUNITY-DWELLING KOREAN OLDER ADULTS

**DOI:** 10.1093/geroni/igz038.2436

**Published:** 2019-11-08

**Authors:** Jinhee Shin, Eunhee Cho

**Affiliations:** College of Nursing, Yonsei University, SOUTH KOREA, Seoul, Korea, Republic of

## Abstract

Objective This study aims to explore predictors to longitudinal decline of cognitive function in old adults in Korean. Methods The data were derived from the information system of the Korean Longitudinal Study of Aging (KLoSA) which performed nationwide aging panel survey for adults aged over 45 years between 2006 and 2016. The sample consisted of 1,262 older adults who completed K-MMSE. Of the total 1,262 participants, 752 had normal cognition, 243 had mild cognitive impairment, and the rest 267 had dementia. Variables from diverse dimensions were derived from the KLoSA. The linear mixed models were used to predict and explain predictors affecting cognitive function decline over time. Results The ADL and IADL, depression, exercise, and social activity were time-varying variables significantly related to the cognitive function of the older adults. Over time, difference in change of the K-MMSE score between three groups was significant. Conclusions This study identified predictors influencing decrease of cognitive function over time in older adults in Korea. Tailored intervention needs to be developed and implemented in order to delay the cognitive function decline. Improving physical function through regular exercise, increasing social activity, and managing depression by early detection and treatment are recommended according to the cognitive function status. Keywords: old adults, cognitive function, K-MMSE, predictor

